# Tissue distribution and subcellular localizations determine *in vivo* functional relationship among prostasin, matriptase, HAI-1, and HAI-2 in human skin

**DOI:** 10.1371/journal.pone.0192632

**Published:** 2018-02-13

**Authors:** Shiao-Pieng Lee, Chen-Yu Kao, Shun-Cheng Chang, Yi-Lin Chiu, Yen-Ju Chen, Ming-Hsing G. Chen, Chun-Chia Chang, Yu-Wen Lin, Chien-Ping Chiang, Jehng-Kang Wang, Chen-Yong Lin, Michael D. Johnson

**Affiliations:** 1 School of Dentistry, National Defense Medical Center, Taipei, Taiwan; 2 Department of Dentistry, Tri-Service General Hospital, Taipei, Taiwan; 3 Graduate Institute of Biomedical Engineering, National Taiwan University of Science and Technology, Taipei, Taiwan; 4 Department of Biomedical Engineering, National Defense Medical Center, Taipei, Taiwan; 5 Division of Plastic Surgery, Department of Surgery, Shuang-Ho Hospital, Taipei, Taiwan; 6 Department of Surgery, School of Medicine, Taipei Medical University, Taipei, Taiwan; 7 Department of Biochemistry National Defense Medical Center, Taipei, Taiwan; 8 Lombardi Comprehensive Cancer Center, Department of Oncology Georgetown University Washington DC, United States of America; 9 School of Medicine, National Defense Medical Center, Taipei, Taiwan; 10 Department of Dermatology, Tri-Service General Hospital, Taipei, Taiwan; University of Minnesota Hormel Institute, UNITED STATES

## Abstract

The membrane-bound serine proteases prostasin and matriptase and the Kunitz-type protease inhibitors HAI-1 and HAI-2 are all expressed in human skin and may form a tightly regulated proteolysis network, contributing to skin pathophysiology. Evidence from other systems, however, suggests that the relationship between matriptase and prostasin and between the proteases and the inhibitors can be context-dependent. In this study the *in vivo* zymogen activation and protease inhibition status of matriptase and prostasin were investigated in the human skin. Immunohistochemistry detected high levels of activated prostasin in the granular layer, but only low levels of activated matriptase restricted to the basal layer. Immunoblot analysis of foreskin lysates confirmed this *in vivo* zymogen activation status and further revealed that HAI-1 but not HAI-2 is the prominent inhibitor for prostasin and matriptase in skin. The zymogen activation status and location of the proteases does not support a close functional relation between matriptase and prostasin in the human skin. The limited role for HAI-2 in the inhibition of matriptase and prostasin is the result of its primarily intracellular localization in basal and spinous layer keratinocytes, which probably prevents the Kunitz inhibitor from interacting with active prostasin or matriptase. In contrast, the cell surface expression of HAI-1 in all viable epidermal layers renders it an effective regulator for matriptase and prostasin. Collectively, our study suggests the importance of tissue distribution and subcellular localization in the functional relationship between proteases and protease inhibitors.

## Introduction

The somewhat contradictory descriptions of the functional relationship between matriptase and prostasin present in the literature provide an interesting example of the diversity and divergence of life in apparently similar systems. Matriptase is a type 2 transmembrane serine protease [1–3] and prostasin is a glycosylphosphatidylinositol (GPI)-anchored or transmembrane serine protease [4, 5]. Matriptase and prostasin can function in concert as a tightly coupled proteolytic cascade [6, 7]. Both proteases are broadly co-expressed in many epithelial tissues in the mouse [8], synthesized and processed through the secretory pathway and anchored on the cell membrane. Matriptase and prostasin resemble one another in that they both possess trypsin-like proteolytic activity, undergoing zymogen activation via cleavage at an Arg residue within an activation motif, and being under the tight control of the hepatocyte growth factor activator inhibitors (HAIs) [9–13]. Almost identical epidermal defects have been observed in the skin of matriptase knockout and prostasin knockout mice [14, 15], and evidence for a functional link between these proteins is further supported by their co-expression in the uppermost viable epidermal layer in mouse skin [6]. The biochemical characteristics of the regulation of proteolytic activity also suggest a functional partnership. Matriptase and prostasin are synthesized as zymogen forms, a shared mechanism among many serine proteases, by which the potential hazards of unfettered proteolytic activity can be moderated by activating the zymogen only at the time and place where the proteolytic activity is needed. Most serine protease zymogens are activated by the action of other proteases that have already been activated, whereas few undergo zymogen activation via autoactivation, an alternative mechanism by which the first active protease in a cascade can be generated in the absence of other active proteases. Matriptase is such a serine protease and acquires proteolytic activity via autoactivation [16]. A functional relationship in which matriptase acts as the upstream activator of the downstream substrate prostasin was initially suggested by the lack of prostasin zymogen activation observed in the skin of matriptase knockout mice [6]. Assessment of the activation state of prostasin in this study depended, however, on being able to discriminate between the zymogen and active forms of prostasin by western blot, based on size (a difference of only 12 amino acids, or less than 5%), raising the possibility that some level of prostasin activation remains. Nevertheless, in HaCaT human keratinocytes, prostasin zymogen activation is induced simultaneously when matriptase zymogen activation is induced, and matriptase is required for the induction of prostasin zymogen activation [7]. Concomitant induction of prostasin and matriptase activation can also be observed in some other epithelial cells [17], indicating that matriptase and prostasin can function as a tightly coupled proteolytic cascade, at least, *in vitro* in cultured cells.

In spite of this well-defined functional relationship, the inverse matriptase and prostasin expression pattern that can be observed through the course of epidermal differentiation [18] suggests that the functional link between the two serine proteases may not be as strong in human skin as has been observed in mouse and cultured human cells. In quiescent human skin, matriptase is primarily expressed by the basal and spinous keratinocytes [7, 18, 19] suggesting a possible role in keratinocyte proliferation and early differentiation [20]. Matriptase expression is negligible in the granular layer of the human skin. A similar matriptase expression profile has been observed during the course of differentiation in the hair follicle and sebaceous gland, both of which are histologically connected with and resemble the epidermis regarding life-long renewal through proliferation and progressive programmed differentiation [18, 21]. The downregulation of matriptase expression associated with increased differentiation is in stark contrast to the differentiation-associated upregulation of prostasin expression that is seen in human epidermis, where prostasin is primarily detected in the keratinocytes of the granular layer with some expression in the upper spinous layers [6]. This inverse trend in the differentiation-state related expression of these proteins raises questions about the validity of the proposed functional link between matriptase and prostasin in the human skin.

Most serine proteases are synthesized as zymogens with limited intrinsic activity. Zymogen activation, which generates the active protease, represents one of the most important mechanisms by which the function and enzymatic activity of proteases can be temporally and spatially regulated. Zymogen activation status can, therefore, also be an important criterion in determining the functional linkage between two serine proteases which are postulated to function as a tightly coupled cascade or which form protein complexes for their mutual zymogen activation [22]. This is particularly true for matriptase, which is almost ubiquitously co-expressed with high levels of HAI-1, the most important endogenous protease inhibitor for matriptase. The result of this pattern of co-expression is that enzymatically active matriptase has a very short half-life, particularly when the enzyme is cell-associated [23]. If matriptase and prostasin indeed function as a tightly coupled protease cascade, or form a reciprocal zymogen activation complex, one might expect that they would exhibit similar dynamics for zymogen activation and inhibition [22]. In the current study, matriptase and prostasin zymogen activation status and the inhibition of these enzymes by HAI-1 and HAI-2 was analyzed and compared in human skin specimens by immunohistochemistry and immunoblot assays. Our data indicate that prostasin exhibits high-level constitutive activation in cells at the later stage of differentiation in the human epidermis, in contrast to matriptase, where zymogen activation is at a much lower level than prostasin and mainly is found on the basal keratinocytes. HAI-1 and not HAI-2 is the main inhibitor of both prostasin and matriptase. Our study provides further evidence that any functional relationship among the two serine proteases and the two Kunitz-type inhibitors is context-dependent and likely due to their respective tissue distributions and subcellular localizations.

## Materials and methods

### Reagents

Alexa Fluor 594 goat anti-mouse IgG and Alexa Fluor 488 phalloidin were obtained from Molecular Probes (ThermoFisher Scientific, Waltham, MA). 5,5’-Dithio-bis-(2-Nitrobenzoic Acid) (DTNB) and 4',6-diamidino-2-phenylindole (DAPI) were obtained from Sigma-Aldrich (St. Louis, MO); Horseradish peroxidase (HRP)-conjugated secondary antibodies were purchased from Kirkegaard & Perry Laboratories (Gaithersburg, MD), Western Lightning Chemiluminescence Reagent Plus was purchased from PerkinElmer Life Sciences (Waltham, MA).

### Cell cultures

HaCaT human keratinocytes (CLS Cell Lines Service GmbH, Eppelheim Germany), were maintained in DMEM supplemented with 10% fetal bovine serum (FBS). The immortalized human mammary epithelial cells 184 A1N4 (a gift from M. R. Stampfer, UC Berkeley) [24] were cultured in a modified Improved Minimum Essential Medium (IMEM) supplemented with 0.5% FBS, 5 μg/ml recombinant human insulin (rh-insulin) (Invitrogen, ThermoFisher Scientific), 5 μg/ml hydrocortisone (Sigma), and 10 ng/ml recombinant human epidermal growth factor (rhEGF) (Promega). The cells were incubated at 37°C in a humidified atmosphere with 5% CO_2_.

### Antibodies

The monoclonal antibody (mAb) M24 for total matriptase, the mAb M69 for activated matriptase, and the mAb M19 for HAI-1 were generated using purified matriptase-HAI-1 complex as the immunogen and characterized previously [7, 9, 25, 26]. The mAb DC16 for HAI-2 was generated using recombinant HAI-2 as the immunogen and characterized previously [11, 27, 28]. The prostasin mAbs YL11 and YL89 were generated using purified prostasin-HAI-1 complex as the immunogen and characterized previously [10, 11, 17].

### Immunohistochemistry

The skin tissue sections were obtained from patients, with written informed consent by the Tri-Service General Hospital (TSGH), National Defense Medical Center under IRB protocol 099-05-019, approved by the TSGH Institutional Review Board (IRB). Immunohistochemical staining was conducted as described in previous studies [18, 19, 29]. Sections of frozen human skin were fixed with formalin and stained using the matriptase mAb M24, the activated matriptase mAb M69, the prostasin mAb YL11, the activated prostasin mAb YL89, the HAI-1 mAb M19, the HAI-2 mAb DC16 or mouse IgG as negative control and followed by the secondary antibody (EnVision+ Dual Link System Peroxidase) (Dako, Glostrup, Denmark). DAB (3,3'-Diaminobenzidine) was used for the detection of positive staining. Cell nuclei were counterstained with hematoxylin. Images were captured using an Olympus AH2 Vanox Microscope System (Olympus, Melville, NY). The specificity of these mAbs and their applications in IHC staining can be found in our previous studies [9, 18, 19, 26, 30, 31].

### Acid-induced zymogen activation of matriptase and prostasin

Human mammary epithelial cells 184 A1N4 and HaCaT human keratinocytes cultured in 60 mm culture dishes were washed with PBS three times and then incubated with 150 mM phosphate buffer pH 6.0 at room temperature for 20 min as previously describe [26, 32]. The cells were then washed with PBS once and lysed in 1% Triton X-100 and 1 mM DTNB in PBS for immunoblot analysis.

### Preparation of human foreskin lysates

Our previous study has shown that the tissue distribution of matriptase in human foreskin is the same as in the adult skin [20]. In order to investigate the zymogen activation state and identify the protein protease inhibitors involved in the control of matriptase and prostasin in human skin, the species of prostasin, matriptase, HAI-1 and HAI-2 in human foreskin were analyzed by western blot. Human foreskin specimens obtained as discarded surgical wastes from the routine circumcision of new born babies under a tissue donation program were generously donated by Dr. Richard Schlegel MedStar Georgetown University Hospital, under the exempted IRB# 2002–021. The IRB Title is: "Collection of Foreskin Following Elective Circumcision of Male Neonates". The foreskin samples were collected with signed Informed Consent Form and provided as anonymous tissues. The specimens were homogenized in PBS using a Kinematica Polytron homogenizer PT2100 (Luzern, Switzerland). The insoluble portion of the homogenate was collected by centrifugation at 20,000 x*g* for 20 min and lysed in RIPA buffer supplemented with the Roche Complete Protease Inhibitor Cocktail (West Sussex, UK). Residual human immunoglobulins in the samples were depleted by incubating the foreskin lysates with Pierce protein A/G agarose (ThermoFisher Scientific).

### Western blotting

Equal amounts of proteins from the cell lysates or tissue lysates were diluted in 5x SDS sample buffer containing no reducing agent and incubated at room temperature for 5 min prior to loading onto the gels. Proteins were resolved by 7.5% SDS-PAGE, transferred to nitrocellulose membranes, and probed with the indicated mAbs. The binding of mAbs was detected using HRP conjugated secondary antibodies, and visualized using Western Lightening Chemiluminescence Reagent Plus (Perkin-Elmer).

## Results

### Generation and characterization of activated prostasin monoclonal antibody

**Fig 1:**
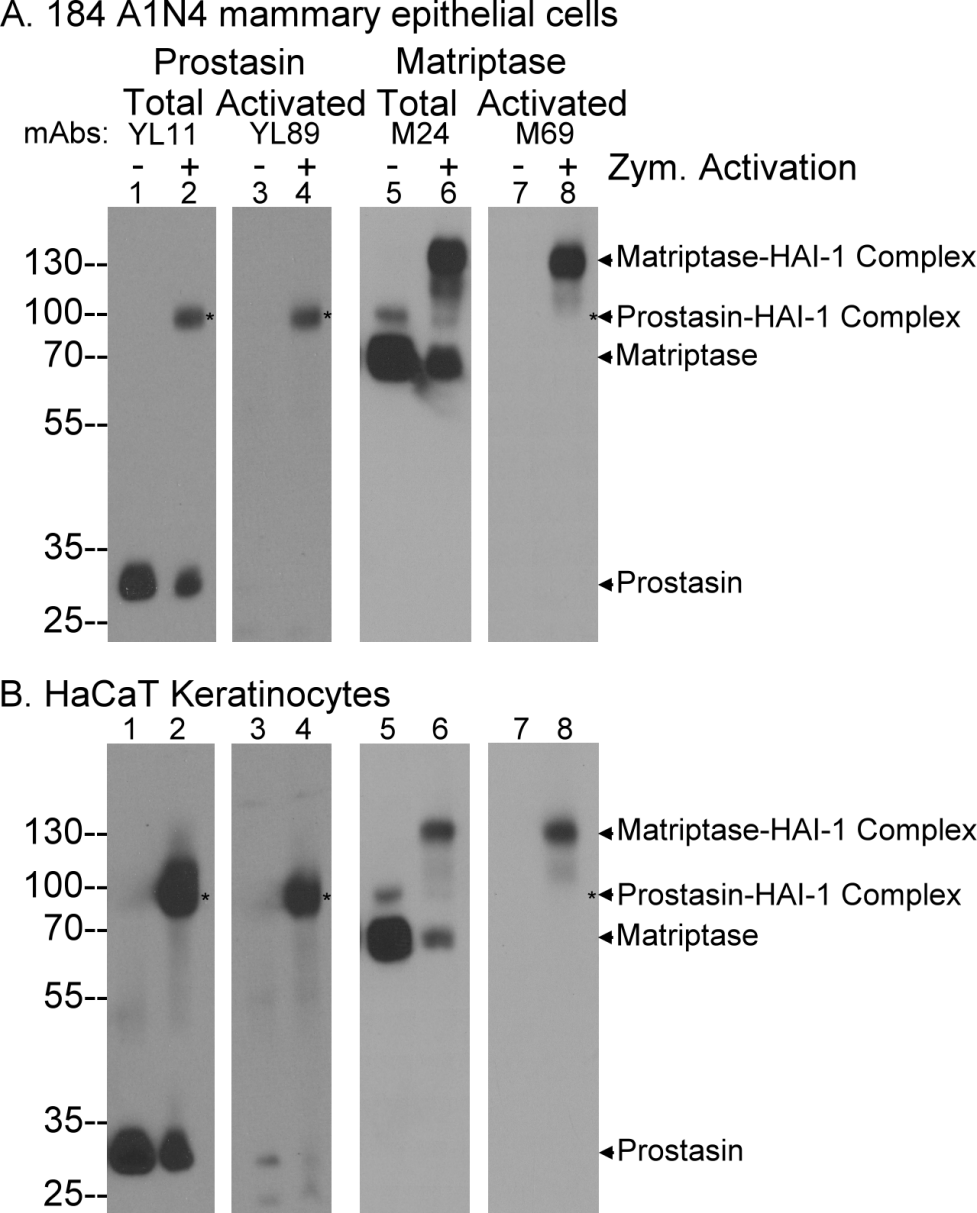
The mAb YL11 selectively targets activated prostasin and not prostasin zymogen. The 184 A1N4 human mammary epithelial cells (A) and the human keratinocytes HaCaT cells (B) were exposed to phosphate buffer saline (PBS) (lanes 1, 3, 5, and 7) as non-activation control or 150 mM phosphate buffer pH 6.0 for 20 min to induce zymogen activation of matriptase and prostasin (lanes 2, 4, 6, and 8). Cell lysates were analyzed by immunoblot for total prostasin using the mAb YL11 (lanes 1 and 2), activated prostasin using the mAb YL89 (lanes 3 and 4), total matriptase using the mAb M24 (lanes 5 and 6), and activated matriptase using the mAb M69 (lanes 7 and 8).

The activated prostasin monoclonal antibody (mAb) YL89 was one of the three prostasin mAbs initially generated using human milk-derived activated prostasin in complex with HAI-1 as the immunogen [10]. The lack of binding of mAb YL89 to prostasin zymogen was noticed when this mAb was used to characterize the zymogen activation and inhibition of prostasin. In 184 A1N4 mammary epithelial cells (Fig 1A) and HaCaT human keratinocytes (Fig 1B), prostasin is expressed mainly in the zymogen form of 28-kDa detected by the total prostasin mAb YL11 (Fig 1, lanes 1). In contrast, the prostasin mAb YL89 did not detect the prostasin zymogen protein band in 184 A1N4 cell lysates (Fig 1A, lane 3), and only a very weak band was seen in this area with HaCaT cell lysates. Upon induction of matriptase and prostasin zymogen activation by transiently exposing the cells to a pH 6.0 buffer, a proportion of the prostasin zymogen was converted to activated prostasin, which forms a stable 100-kDa complex with HAI-1 [7, 17]. The 100-kDa activated prostasin complex can be detected by the total prostasin mAb YL11 (Fig 1, lanes 2, indicated by *) and by the activated prostasin mAb YL89 (Fig 1, lanes 4, indicated by *). The immunoblot analysis in conjunction with the induction of prostasin zymogen activation demonstrates that the YL89 mAb is selective for activated prostasin and does not appear to recognize the prostasin zymogen.

We have previously successfully generated antibodies that are selective for the activated form of a protease using a protease-inhibitor complex as the antigen. In addition to several antibodies that recognize matriptase irrespective of its activation state, immunization of mice with matriptase-HAI-1 complex also allowed us to generate antibodies specific for the activated form of the enzyme which we have characterized extensively in our previous studies [9, 26, 30, 32]. Analyzing the lysates with these antibodies, matriptase zymogen was detected as a 70-kDa protein band in both cell lines (Fig 1, lanes 5). A minor 94-kDa band was also detected which represents the full-length matriptase [16]. The activated matriptase mAb M69 did not detect these two matriptase species (Fig 1, lanes 7). Upon induction of matriptase zymogen activation, a proportion of the matriptase zymogen was converted to activated matriptase in complex with HAI-1. The 120-kDa activated matriptase complex can be detected by the total matriptase mAb M24 (Fig 1, lanes 6) and the activated matriptase mAb M69 (Fig 1, lanes 8). These data are consistent with the known specificities of the two matriptase mAbs.

### Prostasin undergoes constitutive zymogen activation primarily in the cells of the granular layer of human skin

**Fig 2:**
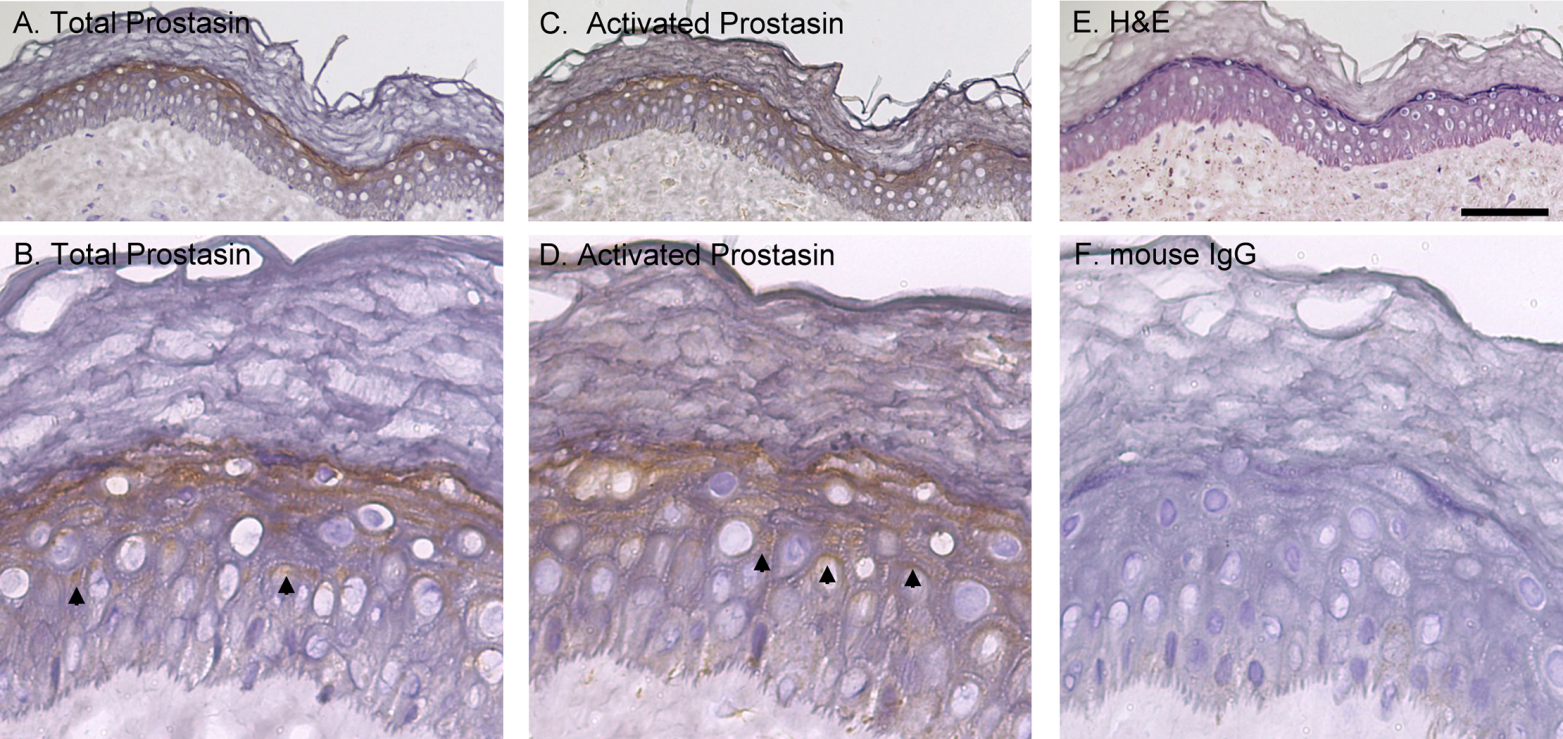
Prostasin is activated at high levels in the later stages of epidermal differentiation in human skin. Sections of human skin, containing the epidermis, were immunostained with the prostasin mAb YL11 (A and B, Total Prostasin), the activated prostasin mAb YL89 (C and D, Activated Prostasin), and mouse IgG as a negative control (F, Mouse IgG). The tissues were counterstained with hematoxylin and eosin (E, H&E). Representative examples of the staining observed are presented. Scale bar: ~100 μm. *n*>20.

With these mAbs, we conducted an immunohistochemical (IHC) study of human skin frozen sections to determine and compare *in vivo* zymogen activation status of prostasin and matriptase. More than 20 skin specimens were analyzed and consistent staining patterns were obtained using these mAbs. Prostasin was detected using the YL11 mAb primarily on cells of the granular layer (Fig 2A). At high magnification, in addition to the intense staining in the granular layer cells right beneath the cornified layer, prostasin was also observed in some spinous layer keratinocytes as polarized patches of staining underneath the intercellular bridges, a pattern characteristic of desmosomes (Fig 2B). The basal cells were devoid of the prostasin staining. The staining pattern for activated prostasin was almost identical to that for the total prostasin with intense staining on the granular cells (Fig 2C, indicated by arrow) as well as polarized patches beneath the desmosomes of spinous cells (Fig 2B and 2D, indicated by arrow). An interesting but potentially important difference in the prostasin staining was noticed in the cornified layer. Faint staining was seen throughout the section in the cornified layer with the activated prostasin antibody, but not with the total prostasin mAb (Fig 2, comparing 2D to 2B). Extensive crosslinking occurs in the cornified layers, which may have interfered with the exposure of the epitopes for both prostasin mAbs though to a great extent for the mAb YL11 than for YL89. If this staining is valid, however, prostasin is also present with, predominantly in the activated form in the cornified keratinocytes in addition to the granular keratinocytes and some spinous keratinocytes. H&E staining of a section is shown in Fig 2E to show the histological structure of the skin, and mouse IgG staining in Fig 2F as a negative control of the IHC staining.

**Fig 3:**
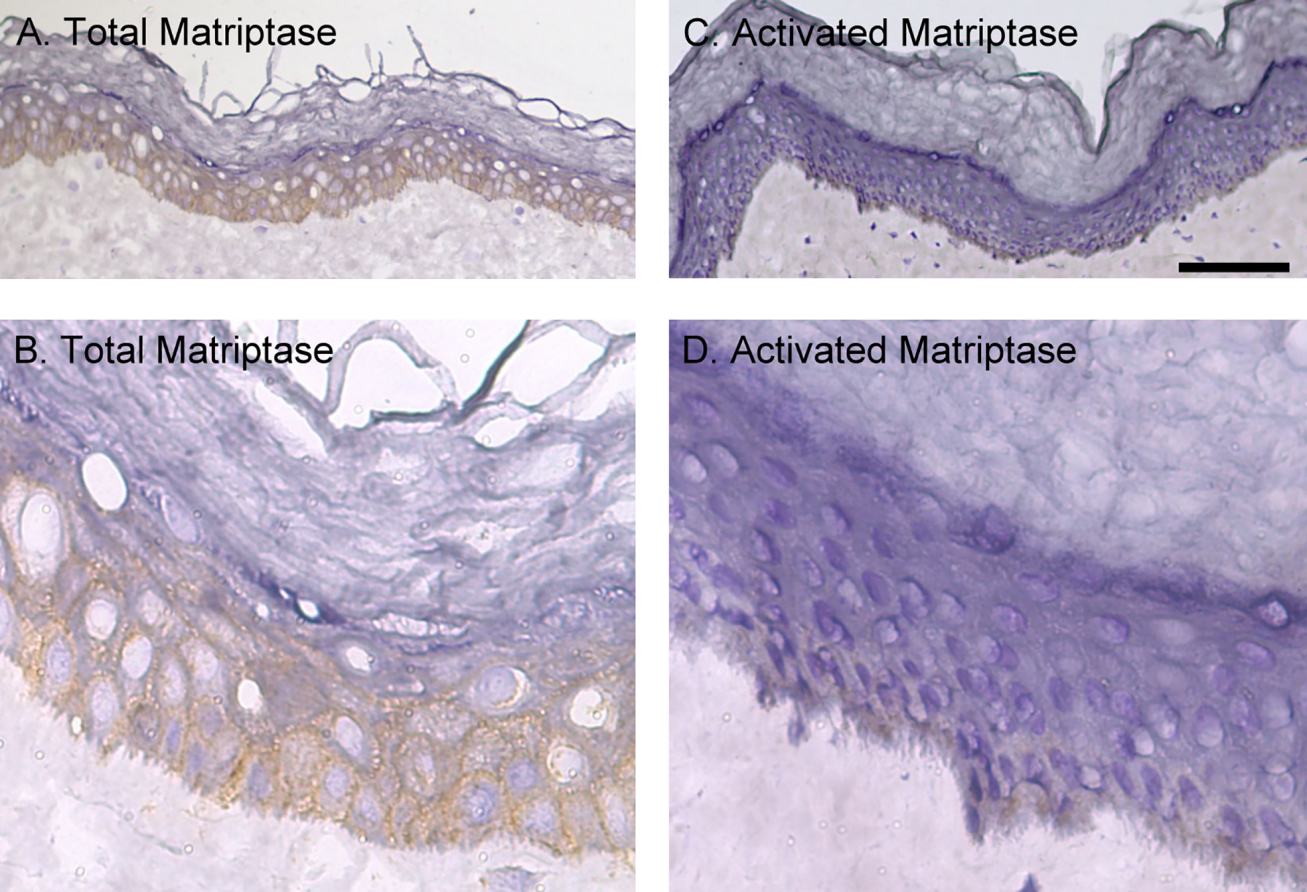
Matriptase is expressed at high levels in human skin, but is largely present in the zymogen form. Sections of human skin were immunostained with the matriptase mAb M24 (A and B, Total Matriptase) and the activated matriptase mAb M69 (C and D, Activated Matriptase). Representative examples of the staining observed are presented. Scale bar: ~100 μm. *n*>20.

**Fig 4:**
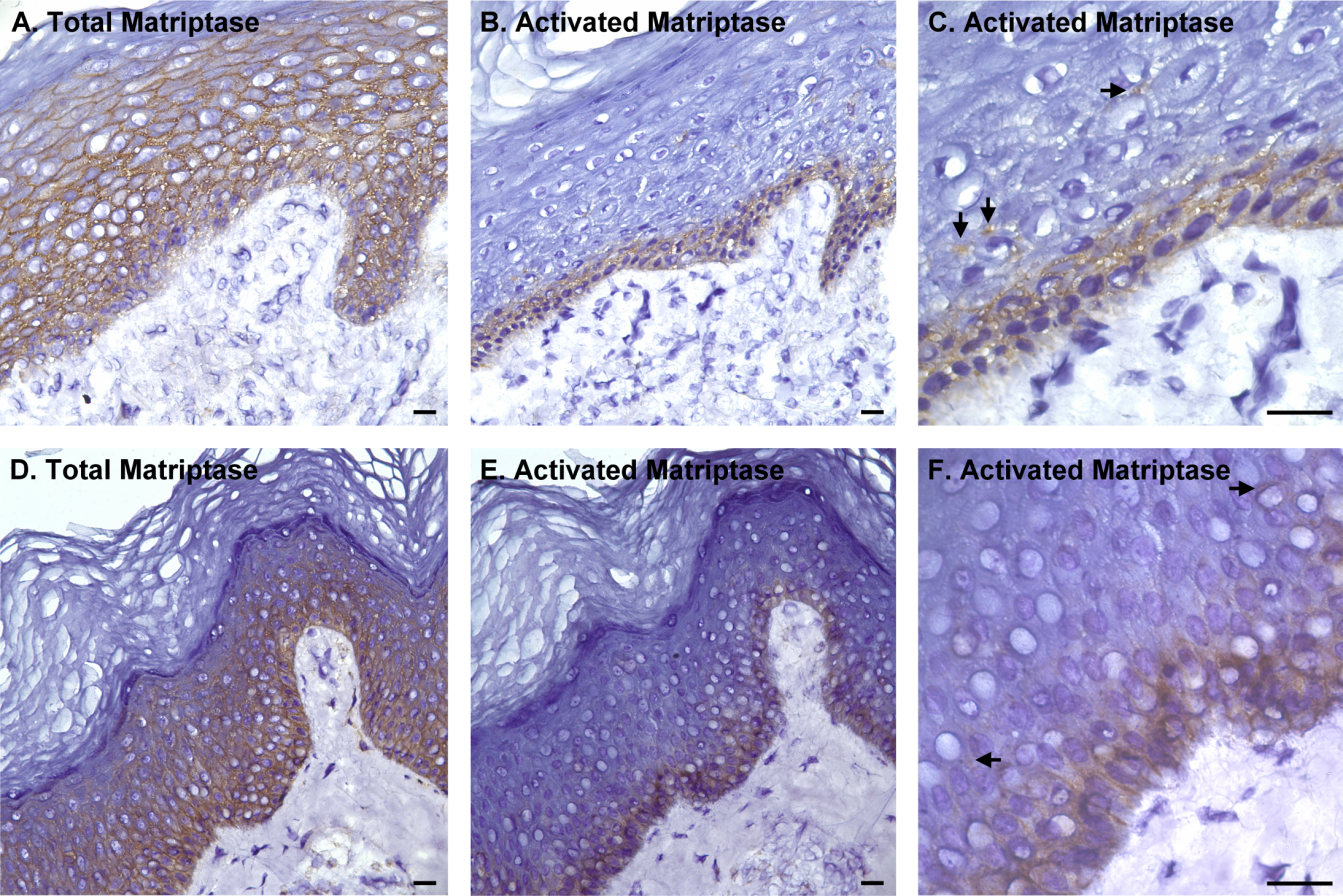
Active matriptase is largely restricted to the basal layer of human skin. Sections of human skin were immunostained with the total matriptase mAb M24 (A and D) and the activated matriptase mAb M69 (B, C, E and F). The cells were counterstained with hematoxylin. Representative examples of the staining observed are presented. Scale bar: 25 μm. *n*>20.

We next examined the tissue distribution and *in vivo* zymogen activation status of matriptase in human skin using sections from the same tissue sample in order to compare the distribution to that for prostasin. In marked contrast to the prostasin staining pattern, no matriptase staining was seen on the cells of the granular layer but was readily detected on the basal and spinous layers cells (Fig 3A). At higher magnification (Fig 3B), matriptase was detected clearly at the intercellular junctions but was not observed at the contact sites between the basal cells and the stroma of the dermis. In contrast to the high levels of total matriptase in the tissues, the level of activated matriptase appears to be very low in the epidermis (Fig 3C). Only very faint staining could be seen on the cells of the basal layer (Fig 3C and 3D). While the signal is generally very low, positive staining for activated matriptase has constantly been observed in a portion of skin specimen. Two examples of samples with much higher levels of activated matriptase staining are shown in Fig 4. In these two specimens, the matriptase expression pattern is confirmed with high level staining in the basal and spinous layers but much lower or undetectable staining in the cells of the granular layer (Fig 4A and 4D). In spite of the high level matriptase expression in the basal and spinous layer cells, staining for activated matriptase was much more restricted and detected predominantly in the basal keratinocytes (Fig 4B and 4E). At higher magnification, activated matriptase was also detected sporadically in a few suprabasal cells (Fig 4C and 4F, as indicated by arrows). The localization of both total and activated matriptase at the intercellular bridges was also clearly seen in these two specimens. Collectively, these IHC staining data suggests that prostasin is expressed mainly by keratinocytes at the later stages of differentiation and appears to be activated at high levels in the cells that express the protease. In contrast, matriptase is expressed by the basal and spinous cells. Matriptase zymogen activation is generally only present at very low levels, but when observed, it is predominantly found in the proliferative basal layer cells and only rarely in differentiating spinous layer cells.

### Prostasin activity is controlled by HAI-1 and not HAI-2

**Fig 5:**
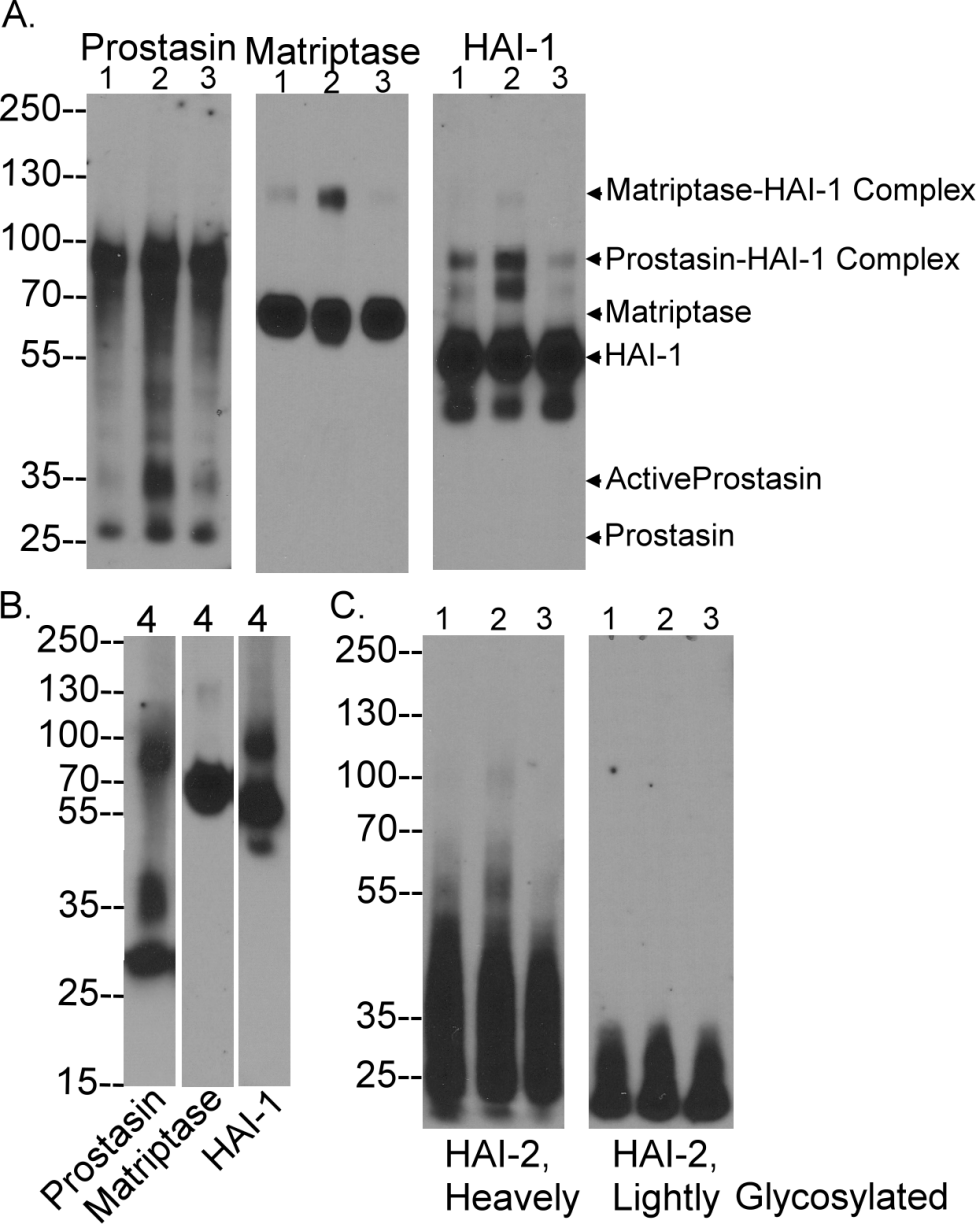
The forms of prostasin, matriptase, HAI-1 and HAI-2 in human foreskin. Human foreskin lysates were prepared from 4 different donors in two batches with specimen 1–3 together and specimen 4 alone. Equal amount of protein from the four tissue lysates were analyzed by immunoblot for prostasin, matriptase, and HAI-1, as indicated (A and B). HAI-2 species were also analyzed in specimens 1–3 (C). The prostasin species include prostasin zymogen, active prostasin, and prostasin complex with HAI-1, as indicated. Matriptase species include matriptase zymogen and matriptase complex with HAI-1, as indicated. HAI-1 species include HAI-1 monomer, HAI-1 complexes with prostasin or matriptase, as indicated. HAI-2 species include the one with heavy N-glycan (C, right panel) and the one with light N-glycan (C, left panel).

The *in vivo* expression and zymogen activation status of prostasin and matriptase were further investigated and confirmed by western blot analysis of lysates prepared from human foreskin tissue from four different neonates. The immunoblot analysis revealed three prostasin species with apparent molecular weights of 28, 35, and 100-kDa. The prostasin zymogen forms a band at 28k-Da and activated prostasin in complex with HAI-1 at 100-kDa (Fig 5A and 5B, Prostasin). The 100-kDa prostasin-HAI-1 complex was also detected by the HAI-1 mAb M19 (Fig 5A and 5B, HAI-1) which also detects the HAI-1 monomer at 55-kDa (Fig 5A and 5B, HAI-1). The 35-kDa prostasin band is likely the free active prostasin, the identification and characterization of which can be found in our previous study [11]. It is worth noting that the western blot was conducted under non-reducing and non-boiled condition, under which the prostasin light chain, which is 12 amino acid long, remains associated with the prostasin heavy chain. The slower migration rate of active prostasin on SDS-PAGE is likely the result of the conformational changes associated with prostasin zymogen activation. The activated to total prostasin ratio varies significantly among the different samples ranging from more than 50% in the specimen #4 to around 90% in the specimens #1–3. The reason for this significant variation remains unclear. Nevertheless, prostasin is activated at high levels in the human skin and HAI-1 is the predominant protease inhibitor of prostasin in human skin.

The high levels of zymogen activation in marked contrast to the situation for matriptase in the skin. Matriptase was detected predominantly in its 70-kDa zymogen form in the four foreskin lysates (Fig 5A and 5B, Matriptase). The amount of 120-kDa activated matriptase in complex with HAI-1 detected by both matriptase and HAI-1 mAbs was almost undetectable in specimens #3 and #4, very low in specimen #1, and modest in specimen #2 (Fig 5A and 5B, Matriptase and HAI-1). The western blot data for the level of activated matriptase in the foreskin lysates is consistent with the IHC data. The predominance of the zymogen form suggests that matriptase activation is under much tighter control *in vivo* in human skin than prostasin. Like prostasin, active matriptase is inhibited by forming a complex with HAI-1. It is worth noting that in addition to being detected as a monomer (55-kDa) and in the complexes with activated matriptase (120-kDa) or with activated prostasin (100-kDa), HAI-1 was also detected as 80-kDa and 50-kDa bands, which could represent the degraded products of HAI-1 or HAI-1 complexes.

Although HAI-2 has been identified as a major protease inhibitor of prostasin in human intestinal tissues and cultured colon carcinoma cells [11] and as an important inhibitor of matriptase in breast cancer cells[27], the vast majority of the HAI-2 was detected in its monomer forms in the four foreskin lysate samples. HAI-2 can be expressed with extensive or light N-glycosylation and the two HAI-2 species can be distinguished through the use of two different HAI-2 mAbs [28]. Both HAI-2 species appear to be present in the lysates almost entirely in their monomer forms (Fig 5C). Very low levels of HAI-2 complexes with apparent sizes of 100-kDa and 55-kDa were seen in the specimen #2. The 100-kDa HAI-2 species could be a HAI-2-matriptase complex, and the 55-kDa species could be a prostasin-HAI-2 complex, but the very low levels of these HAI-2 complexes suggest that HAI-2 only plays a limited role in the control of matriptase and prostasin activity in human skin.

### The different roles of HAI-2 and HAI-1 in the inhibition of prostasin and matriptase are correlated with their different tissue distribution and subcellular localization

**Fig 6:**
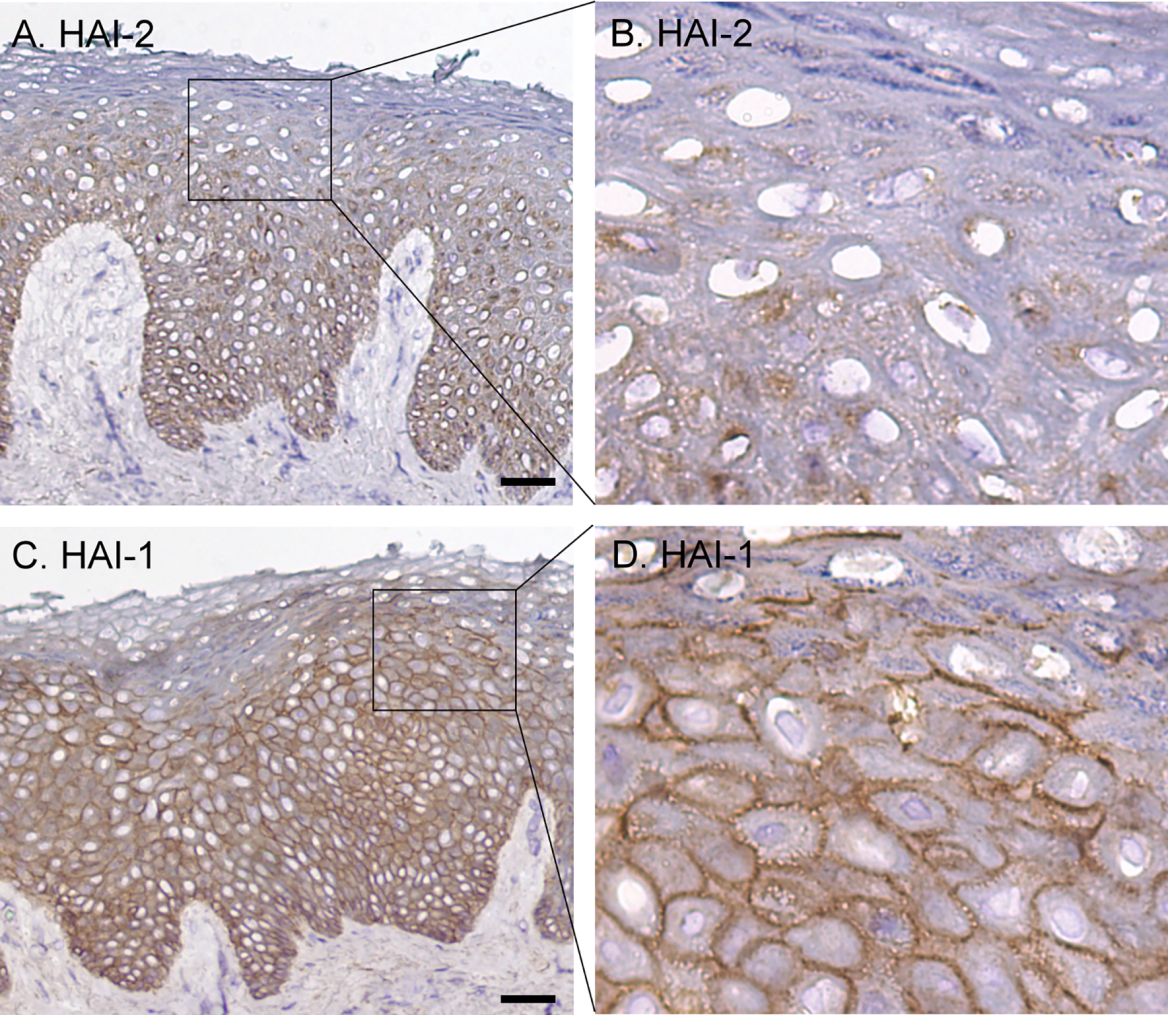
Expression pattern and subcellular localization of HAI-1 and HAI-2 in human skin. Sections of human skin were immunostained with the HAI-2 mAb DC16 (A and B), the HAI-1 mAb M19 (C and D), and mouse IgG as negative control (data not shown). The cells were counterstained with hematoxylin. Representative examples of the staining observed are presented. Scale bar: 50 μm *n*>20.

The limited role of HAI-2 in the control of matriptase and prostasin could be the result of its tissue distribution and subcellular localization. To investigate this possibility, IHC analysis of human skin sections was conducted using the HAI-1 mAb DC16. HAI-2 staining was observed in the basal and spinous layers but not in the granular layers (Fig 6A). At higher magnification (Fig 6B), HAI-2 staining was clearly seen as intracellular patches predominantly localized to one side of nuclei. This tissue distribution suggests that HAI-2 like matriptase is more likely to contributes to human skin biology of the proliferating and differentiating cells rather than in the differentiated cells. This expression pattern is also consistent with HAI-2 playing a less important role as a protease inhibitor for regulating prostasin proteolytic activity. While HAI-2 and matriptase are expressed with a similar pattern over the course of epidermal differentiation, the limited importance of HAI-2 as a matriptase inhibitor suggested by the immunoblot data, could be explained by the largely intracellular localization of HAI-2 versus largely cell surface localization of matriptase.

In contrast to the intracellular localization of HAI-2, HAI-1 was detected on the surface of the keratinocytes in all three viable epidermal layers (Fig 6C). At higher magnification, HAI-1 staining was seen on the intercellular bridges as discontinuous bars between two neighboring cells. The surface staining intensity for HAI-1 appears to increase along with epidermal differentiation with elevated staining more frequently seen on the granular keratinocytes (Fig 6D). The widespread expression on the cell surface in all three epidermal layers provides HAI-1 with direct access not only to matriptase but also prostasin, consistent with the immunoblot data and its apparent role in the control of matriptase and prostasin.

**Fig 7:**
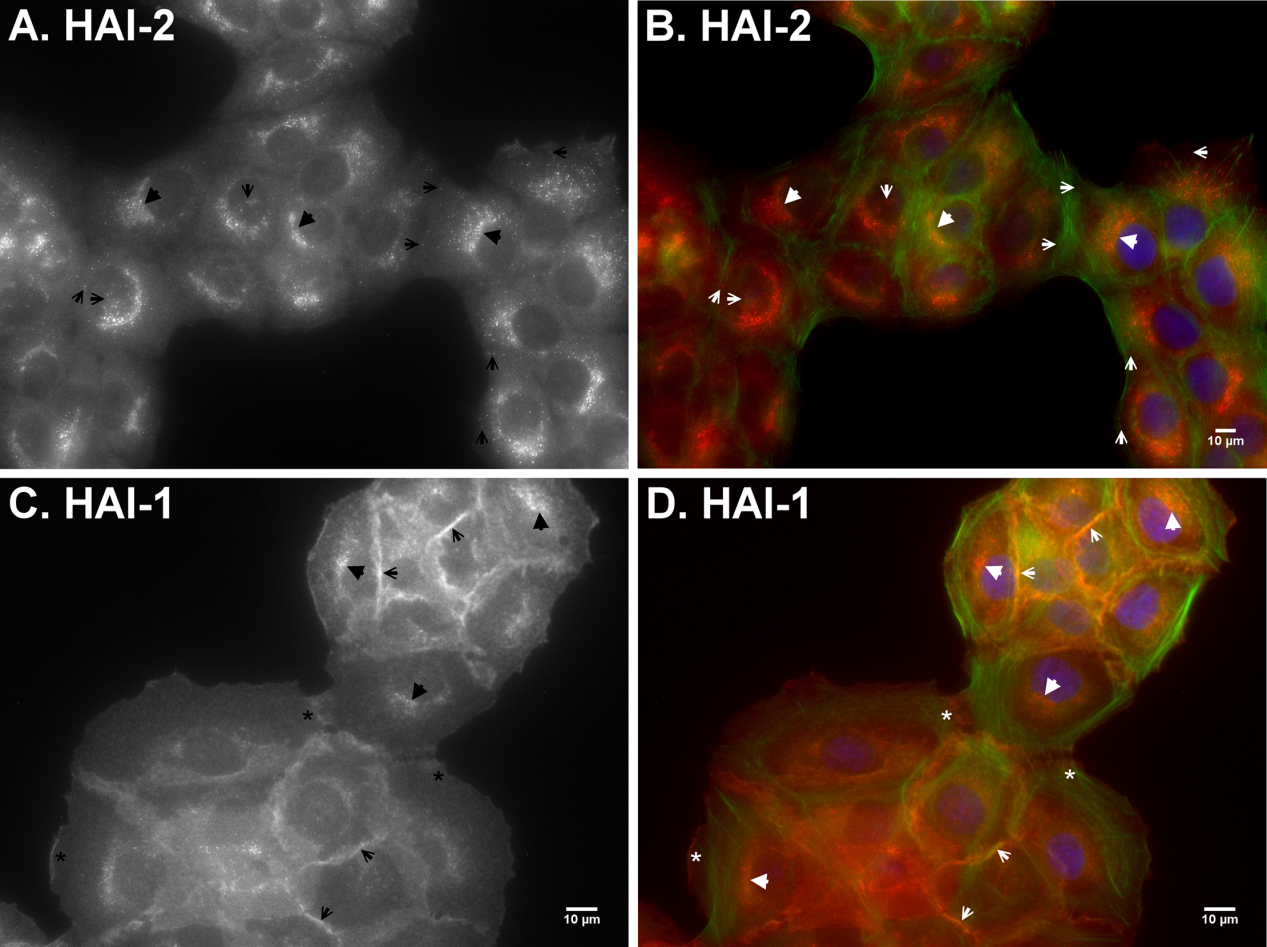
HAI-1 is primarily targeted to the intercellular contacts and HAI-2 largely remains inside HaCaT human keratinocytes. The subcellular localizations of HAI-2 (A and B) and HAI-1 (C and D) in HaCaT human keratinocytes were analyzed by indirect immunofluorescent staining with the HAI-2-specific mAb DC16 and HAI-1-specific mAb M19, followed by Alexa 594-labelled anti-mouse IgG. The cells were also stained for F-actin using Alexa 488-labelled phalloidin (B and D, green) and nuclei using DAPI (B and D, blue), as counterstain. The staining is presented as black and white images (A and C) and merged false-color images (B and D). The staining of matriptase at different types of cell-cell contacts are as indicated. Scale bar: 10 μm.

The different subcellular localizations of HAI-1 and HAI-2 can also be observed *in vitro* using HaCaT human keratinocytes (Fig 7). Indirect immunofluorescent staining of the human keratinocytes was performed with HAI-2 mAb DC16 (Fig 7A and 7B) and HAI-1 mAb M19 (Fig 7C and 7D). The immunostaining revealed HAI-2 primarily as perinuclear patches accumulated on one side of nuclei (Fig 7A, indicated by arrow heads). HAI-2 staining was also seen as small spheres which had broader distribution from the top of nuclei to the periphery of cells (Fig 7A, indicated by arrows). In the merged image also showing actin staining in red, and the nuclei in blue (Fig 7B), the polarized perinuclear staining and the broader spheres staining can be clearly seen (red). A polarized perinuclear staining signal was also seen for HAI-1 (Fig 7C and 7D, indicated by arrow heads), however, the majority of the HAI-1 staining was observed at the intercellular contacts (Fig 7C and 7D, indicated by arrows). In addition, HAI-1 was also detected on the cell periphery in the absence of contact with other cells or where the intercellular junctions were forming or dissociating (Fig 7C and 7D, indicated by *). The perinuclear staining pattern for HAI-2 and HAI-1 is consistent with their synthesis and maturation through the secretory pathway. HAI-1 appears to be targeted to cell surface either at intercellular junctions or on the free cell surface. In contrast, HAI-2 appears to be targeted to intracellular vesicle-like structures. The IHC and immunofluorescent staining demonstrate distinct subcellular targeting of HAI-2 versus HAI-1.

## Discussion

The tissue distribution, the *in vivo* zymogen activation status, the *in vivo* and *in vitro* subcellular localization, and the level of protease-protease inhibitor complexes demonstrated in the current study provide important information that informs an assessment of the putative functional relationship between matriptase and prostasin and between the two proteases and HAIs in human skin. A close functional linkage between the two serine proteases and among these proteases and protease inhibitors have been suggested by an array of previous studies using a variety of model systems. The proposed functional relationships include one involving a reciprocal activator-substrate interaction between matriptase and prostasin and another involving potent inhibition of matriptase and prostasin by HAI-1 and HAI-2. Our previous studies, however, suggested that though they may be correct in some contexts these proposed functional linkages can not be generalized and are somewhat tissue-selective or context-dependent [11, 17, 18]. In the current study, the context-dependent nature of any functional relationship is further supported by the distinct pattern of zymogen activation observed in human skin samples, in which prostasin is activated at high levels primarily in the granular layer whereas matriptase zymogen activation occurs at much lower levels predominantly in the cells of the basal layer. Similarly, although HAI-2 has been identified as a relevant matriptase inhibitor in breast cancer cells, and prostasin inhibitor in human enterocytes and colon carcinoma cells, HAI-2 does not appear to be an important protease inhibitor for matriptase or prostasin in human skin, in which the tissue distribution and subcellular localization of HAI-2 suggests that HAI-2 has limited access to active matriptase or prostasin. Although mouse HAI-2 may also play no role in the control of matriptase and prostasin in mouse epidermis, the mechanism responsible for the lack of a functional interactions appears to be different. HAI-2 is apparently not expressed in mouse skin [33], a piece of evidence that further underlines the genuine physiology differences between human skin and mouse skin.

The tight control of matriptase zymogen activation in normal skin with activation seems to be limited to the basal layer, suggests its role may normally be limited to this important compartment. Increased matriptase zymogen activation has, however, been observed in a variety of distinct skin diseases, particularly those in which inflammation is involved [19]. Inflammation-induced oxidative stress is likely to be the underlying mechanism for the increased matriptase zymogen activation as matriptase zymogen activation has been shown to be induced under oxidized conditions [34]. Recently, plasminogen has been identified as another inducer of matriptase zymogen activation selectively in differentiating primary human keratinocyte [35]. Matriptase proteolytic activity could, thus, be significantly induced with the influx of plasminogen with blood and the disruption of calcium gradient when cutaneous wound occurs. The resultant active matriptase in turn accelerates plasmin generation via the activation of plasminogen activator. Given the critical role of plasmin in wound healing, the induced matriptase proteolysis might be involved in the epidermal response to skin wounding and subsequent healing. Matriptase could, therefore, exert its function when human skin is under insults and needs rapid response and repair. This further supports the hypothesis that matriptase functions as an “emergency” protease, consistent with the unusual feature of matriptase zymogen activation, which can be rapidly and/or robustly induced.

The regulated nature of matriptase zymogen activation and its confinement primary to the basal keratinocytes is in stark contrast to the high-level activation of prostasin that is predominantly observed in cells in the later stages of epidermal differentiation. The high levels of zymogen activation suggests that prostasin proteolytic activity is likely constitutive and that it participates in the maintenance of the epidermal renewal processes in quiescent human skin. The primary expression of this protease in the granular layer further suggests that prostasin proteolytic activity is involved in the late stages of epidermal differentiation, such as the processes involved in epidermal barrier formation.

The detection of high levels of activated prostasin suggests that prostasin undergoes significant zymogen activation in quiescent human skin. In human intestine, the majority of prostasin was also detected in the activated form in complexes with HAI-1 or HAI-2 [11]. Furthermore, prostasin was initially purified from human semen as the enzymatically active form [36] and recently from human milk as the activated form in complexes with HAI-1 or HAI-2 [10]. These data strongly support that significant prostasin zymogen activation could be a widespread phenomenon *in vivo* in human. The significant zymogen activation in conjunction with the lack of another functional protein domain suggests that prostasin’s physiological function, at least in the intestines, skin, prostate, and lactating mammary gland, is likely dependent on its proteolytic activity. The significant level of prostasin zymogen activation detected *in vivo* is at odds with the recent suggestion that prostasin zymogen and not prostasin enzymatic activity participates in the activation of several identified substrates and its biological functions [22, 37–40]. If the biological function of prostasin depends on prostasin proteolytic activity, the primary regulatory mechanism is the process of zymogen activation, by which prostasin gains this enzymatic activity via localized conformational changes leading to the formation of substrate binding pocket following cleavage at Arg-44. If, however, the biological function of prostasin is carried out by its zymogen form, then modulation of expression levels would be the only mechanism to regulate prostasin biological function other than some putative post-translation modification that has yet to be described. Since prostasin does undergo significant zymogen activation *in vivo*, prostasin enzymatic activity, the mechanism governing prostasin zymogen activation, and the inhibition of active prostasin by the HAIs represent important potential mechanisms for the regulation and subsequently the biological function of prostasin *in vivo*. These conventional protease regulatory mechanisms and the proteolytic activity are likely not applicable to the suggested biological functions of prostasin zymogen. If prostasin zymogen is the functional form for its biological functions, what would be the structural basis for prostasin regulation and functions?
