# Correction: Tissue distribution and subcellular localizations determine *in vivo* functional relationship among prostasin, matriptase, HAI-1, and HAI-2 in human skin

**DOI:** 10.1371/journal.pone.0198569

**Published:** 2018-05-31

**Authors:** Shiao-Pieng Lee, Chen-Yu Kao, Shun-Cheng Chang, Yi-Lin Chiu, Yen-Ju Chen, Ming-Hsing G. Chen, Chun-Chia Chang, Yu-Wen Lin, Chien-Ping Chiang, Jehng- Kang Wang, Chen-Yong Lin, Michael D. Johnson

The affiliation for the eleventh and twelfth authors is incorrect. Chen-Yong Lin and Michael D. Johnson are not affiliated with #10, but with #8, Lombardi Comprehensive Cancer Center, Department of Oncology Georgetown University Washington DC, United States of America. There is also an error in affiliation #6 for author Shun-Cheng Chang. Affiliation #6 should be Department of Surgery, School of Medicine, College of Medicine, Taipei Medical University, Taipei, Taiwan.
